# Dose-Dependent Pharmacokinetics of Tofacitinib in Rats: Influence of Hepatic and Intestinal First-Pass Metabolism

**DOI:** 10.3390/pharmaceutics11070318

**Published:** 2019-07-05

**Authors:** Ji Sang Lee, So Hee Kim

**Affiliations:** College of Pharmacy and Research Institute of Pharmaceutical Science and Technology, Ajou University, 206 Worldcup-ro, Yeongtong-gu, Suwon 16499, Korea

**Keywords:** tofacitinib, dose-dependent pharmacokinetics, hepatic and intestinal first-pass effect, rats

## Abstract

This study investigated the pharmacokinetics of tofacitinib in rats and the effects of first-pass metabolism on tofacitinib pharmacokinetics. Intravenous administration of 5, 10, 20, and 50 mg/kg tofacitinib showed that the dose-normalized area under the plasma concentration-time curve from time zero to infinity (AUC) was significantly higher at 50 mg/kg than at lower doses, a difference possibly due to saturation of the hepatic metabolism of tofacitinib. Oral administration of 10, 20, 50, and 100 mg/kg tofacitinib showed that the dose-normalized AUC was significantly higher at 100 mg/kg than at lower doses, a difference possibly due to saturation of the intestinal metabolism of tofacitinib. Following oral administration of 10 mg/kg tofacitinib, the unabsorbed fraction from the rat intestine was 3.16% and the bioavailability (*F*) was 29.1%. The AUC was significantly lower (49.3%) after intraduodenal, compared to intraportal, administration, but did not differ between intragastric and intraduodenal administration, suggesting that approximately 46.1% of orally administered tofacitinib was metabolized through an intestinal first-pass effect. The AUC was also significantly lower (42%) after intraportal, compared to intravenous, administration, suggesting that the hepatic first-pass effect on tofacitinib after entering the portal vein was approximately 21.3% of the oral dose. Taken together, these findings suggest that the low *F* of tofacitinib is due primarily to intestinal first-pass metabolism.

## 1. Introduction

**Figure 1:**
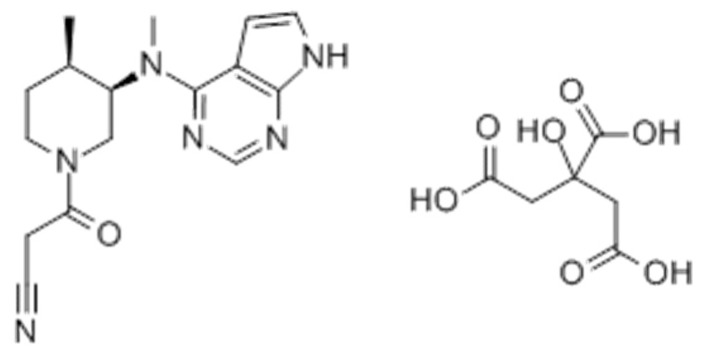
Structure of tofacitinib citrate.

Tofacitinib (3-[(3*R*,4*R*)-4-methyl-3-[methyl-(7*H*-pyrrolo[2,3-day]pyrimidin-4-l)amino]piperidin-1-yl]-3-oxopropanenitril, Figure 1) potently and selectively inhibits Janus kinases (JAK) 1 and 3 through blocking the signal transducer and activator of transcription 1 (STAT1) signaling pathway, thereby suppressing the production of inflammatory mediators, including interleukins-2, -4, -7, -9, -15 and -21 [1,2,3]. These findings led to the use of tofacitinib in the treatment of diseases involving the immune system and to its approval for the treatment of rheumatoid arthritis, particularly in patients intolerant to methotrexate therapy [4]. Tofacitinib was also approved by the US Food and Drug Administration in 2018 for the treatment of moderate to severe ulcerative colitis [5], making it the first oral JAK inhibitor for chronic use in patients with ulcerative colitis [6].

Oral administration of 10 mg tofacitinib to healthy volunteers resulted in an absolute oral bioavailability (*F*) of approximately 74% [7]. Pharmacokinetic analysis showed that its volume of distribution was 87 L, and its terminal half-life was 3.2 h [3,7,8]. Studies found that 40% of oral tofacitinib bound to plasma proteins [3,8], and that approximately 70% of eliminated tofacitinib was excreted in urine after being metabolized, with the remaining 30% eliminated unmetabolized through the kidneys [3,7,8]. Tofacitinib is metabolized through hepatic oxidation and *N*-demethylation, primarily by cytochrome P450 (CYP) 3A4, but also by CYP2C19 and glucuronide conjugation [3]. Although the dose-dependent pharmacokinetics of tofacitinib have been described in humans [7,8], the mechanisms underlying its incomplete absorption have not yet been characterized. However, it is difficult to get the information from clinical settings for evaluating the mechanisms of its incomplete absorption. Instead, using a rat model has been considered, but the basic pharmacokinetic characteristics of tofacitinib in rats has not been thoroughly investigated yet.

The present study assessed the dose-dependent pharmacokinetics of tofacitinib administered both intravenously and orally in rats by evaluating the total area under the plasma concentration–time curve from time zero to infinity (AUC). This study also investigated the effects of first-pass hepatic, gastric, and intestinal metabolism on tofacitinib administered to rats intravenously, intraportally, intragastrically, and intraduodenally. Furthermore, biliary excretion and tissue distribution of intravenously administered tofacitinib were evaluated in rats.

## 2. Materials and Methods

### 2.1. Chemicals

Tofacitinib citrate and hydrocortisone, the internal standard for high-performance liquid chromatography (HPLC) analysis, were obtained from Sigma-Aldrich (St. Louis, MO, USA), and ethyl acetate was from J.T. Baker (Phillipsburg, NJ, USA). Heparin and 0.9% NaCl-injectable solution were purchased from JW Pharmaceutical Corporation (Seoul, Korea), and β-cyclodextrin was from Wako (Osaka, Japan). All other chemicals were HPLC grade and were used without further purification.

### 2.2. Animals

Male Sprague-Dawley rats, aged 7–8 weeks and weighing 240–260 g, were purchased from OrientBio Korea (Seongnam, Korea), housed individually in a clean room, and maintained at a temperature of 22 ± 1 °C, with 12-h light (07:00–19:00) and 12-h dark (19:00–07:00) cycles at a relative humidity of 50 ± 5% with air filtration (Laboratory Animal Research Center of Ajou University Medical Center, Suwon, Korea). The rats had access to food (Purina Korea, Pyeongtaek, Korea) and water *ad libitum*. All experimental procedures and protocols were reviewed and approved by the Institutional Animal Care and Use Committee (IACUC No. 2017-0074, 2018) of the Laboratory Animal Research Center of Ajou University Medical Center.

### 2.3. Estimation of the Appropriate Number of Animals

The appropriate number of animals in each group (*n*) and total number of animals in each experimental setting (*N*) were calculated based on the statistics [9]. For statistical analysis, the degree of freedom should range from 10 to 20. The minimum and maximum number of animals in each group were calculated as:

Minimum *n* = 10/*k* + 1 (1)

Maximum *n* = 20/*k* + 1 (2)

where *k* is the number of groups in each setting. The minimum and maximum *n* were rounded up and down, respectively. The total minimum and maximum *N* required were minimum *n* multiplied by *k* and maximum *n* multiplied by *k*, respectively.

### 2.4. Intravenus and Oral Administration of Tofacitinib

The pretreatment and surgical procedures for oral and intravenous administration were similar to those described previously [10,11]. For oral administration, the rats were fasted overnight with free access to water. The rats were anesthetized with ketamine (200 mg/kg), and their carotid arteries were cannulated using polyethylene tubing (Clay Adams, Parsippany, NJ, USA) for blood sampling. For intravenous administration, the rats were anesthetized with ketamine (200 mg/kg), and their jugular veins and carotid arteries were cannulated for drug administration and blood sampling, respectively. Rats were allowed to recover for 4–5 h after surgical procedures. The rats were not restrained during the experimental period and had free access to water and food.

For intravenous administration, tofacitinib, dissolved in 0.9% NaCl-injectable solution containing 0.5% β-cyclodextrin, was injected via the jugular vein for 1 min at doses of 5 (*n* = 9), 10 (*n* = 8), 20 (*n* = 7), and 50 (*n* = 7) mg/kg. Blood samples (110–220 μL) were collected via the carotid artery at times 0 (prior to drug administration), 1 (at the end of drug infusion), 5, 15, 30, 45, 60, 90, 120, 180, 240, 360, 480, and 600 min. The total amount of blood collected from each rat did not exceed 10% of the total blood volume during the entire experimental period so as not to alter the pharmacokinetics and physiological functions. These blood samples were immediately centrifuged at 8000× *g* for 10 min, and plasma was collected and stored at −80 °C until HPLC analysis of tofacitinib [12]. To prevent blood clotting, 0.3 mL of heparinized 0.9% NaCl-injectable solution (20 IU/mL) was immediately injected into the carotid artery after each blood sampling. Urine samples were collected over 24 h; in addition, each metabolic cage was rinsed with 20 mL of distilled water 24 h after drug administration, and the rinses were combined with their corresponding 24-h urine samples. The volumes of the combined urine samples were measured, and two 100 µL aliquots of each were stored at −80 °C until HPLC analysis of tofacitinib [12]. At 24-h, each rat was exsanguinated, followed by cervical dislocation. The abdomen of each rat was opened and the entire gastrointestinal tract, including its contents and feces, was removed, transferred to a beaker containing 50 mL methanol, and cut into small pieces using scissors. The contents of each beaker were stirred manually with a glass rod for 1 min, and two 100 μL aliquots of each supernatant were collected and stored at −80 °C until HPLC analysis of tofacitinib [12].

For oral administration, approximately 1.0 mL tofacitinib was administered to rats at doses of 10 (*n* = 7), 20 (*n* = 8), 50 (*n* = 9), and 100 (*n* = 7) mg/kg. Blood samples (110–220 μL) were collected via the carotid artery at times 0 (prior to drug administration), 5, 15, 30, 45, 60, 90, 120, 180, 240, 360, 480, 600, and 720 min. Urine and gastrointestinal tract samples were also obtained over 24 h were processed as described above for the corresponding samples collected after intravenous administration.

### 2.5. Hepatic First-Pass Effects of Tofacitinib

The carotid artery and jugular vein were handled as described previously [10,11]. In addition, the vein from the cecum was cannulated and the cannula was pushed forward about 4 cm toward the liver through the portal vein to minimize the damage from blood flowing into the portal vein [13,14]. Using a peristaltic pump (BT-300CA, JIH Pump, Chongqing, China), 10 mg/kg tofacitinib was infused over 30 min into the jugular vein (*n* = 7) and an equal volume of 0.9% NaCl-injectable solution containing 0.5% β-cyclodextrin was infused simultaneously over 30-min into the portal vein. In another group of rats, 10 mg/kg tofacitinib was infused over 30 min into the portal vein (*n* = 7) and an equal volume of 0.9% NaCl-injectable solution containing 0.5% β-cyclodextrin was infused simultaneously over 30 min into the jugular vein. Blood samples were collected from the carotid artery at times 0, 15, 30 (at the end of the infusion), 31, 35, 45, 60, 90, 150, 210, 270, 390, 510, and 630 min, with all sample collection and processing procedures identical to those described above.

### 2.6. Gastric and Intestinal First-Pass Effect of Tofacitinib

Rats were fasted overnight with free access to water. Cannulae were inserted into the carotid artery and the cecal vein [13,14]. Using the peristaltic pump, 10 mg/kg tofacitinib was infused over 30 min into the portal vein (*n* = 6), and equal volumes of 0.9% NaCl-injectable solution containing 0.5% β-cyclodextrin were instilled into both the stomach and duodenum using a 23-gauge needle. In addition, 10 mg/kg tofacitinib was instilled into the duodenum (*n* = 5), and equal volumes of 0.9% NaCl-injectable solution containing 0.5% β-cyclodextrin were instilled into the stomach and infused via the portal vein over 30 min. Furthermore, 10 mg/kg tofacitinib was instilled into the stomach (*n* = 5), and equal volumes of 0.9% NaCl-injectable solution containing 0.5% β-cyclodextrin were instilled into the duodenum and infused via the portal vein over 30 min. Blood samples were collected from the carotid artery at times 0, 15, 30, 31, 35, 45, 90, 150, 270, 390, 510, 630, and 750 min after the start of intraportal infusion of tofacitinib, and at times 0, 5, 15, 30, 60, 120, 180, 240, 360, 480, 600, and 720 min after intragastric and intraduodenal instillations of the drug. All sample collection and processing procedures were identical to those described above.

### 2.7. Tissue Distribution of Tofacitinib

Rats were handled and processed as described previously [10]. Tofacitinib (10 mg/kg) was administered intravenously to rats for 1 min. After 30 min and 2 h (*n* = 4 each), as much blood as possible was collected from the carotid artery. Blood samples were immediately centrifuged, and plasma was collected. The rats were sacrificed by cervical dislocation, approximately 1 g of each brain, fat, heart, kidney, large intestine, liver, lung, mesentery, muscle, small intestine, spleen, and stomach was removed, rinsed with phosphate-buffered solution (pH 7.4), and blotted dry with paper towels to remove any remaining blood. Each tissue sample was added to four volumes of homogenizing buffer, homogenized using a tissue homogenizer (T25 Ultra-Turrax, IKA Labortechnik, Staufen, Germany) and centrifuged at 8000 × g for 10 min. A 100 μL aliquot of each supernatant was collected and stored at −80 °C until HPLC analysis of tofacitinib [12].

### 2.8. Biliary Excretion of Tofacitinib

The jugular vein of each rat was cannulated. The abdomen was opened and the bile duct was cannulated with polyethylene tubing [14]. The incision was closed with surgical sutures and each rat was kept warm under an electric light. Each rat was maintained in the supine position during the entire experiment. Tofacitinib (10 mg/kg) was infused for 1 min via the jugular vein (*n* = 3). Bile samples were collected over various time periods, from 0–1, 1–2, 2–4, 4–6, and 6–24 h. The volume of each bile sample was measured, and an aliquot of each was stored at −80 °C until HPLC analysis of tofacitinib [12].

### 2.9. HPLC Analysis of Tofacitinib

Two microliters of hydrocortisone (5 mg/mL) and 40 μL of 20% ammonia solution were added to 100 μL aliquots of biological samples and vortex-mixed for 30 s using a vortex mixer. Each solution was extracted with 1.5 mL of ethyl acetate by centrifugation at 12,000 rpm for 5 min. The organic layer was collected and dried (Dry Thermobath, Eyela, Tokyo, Japan) under a gentle stream of nitrogen gas at 40 °C. The samples were reconstituted in 130 μL of 20% acetonitrile, and 50 μL of resuspended samples were analyzed by HPLC [12].

The concentrations of tofacitinib in the prepared biological samples were determined using a Shimadzu Prominence LC-20A HPLC system (Kyoto, Japan), consisting of a pump (LC-20A), an auto-sampler (SIL-20A), a column oven, and a detector (SPD-20A/20AD), controlled by the CBM-20A system controller. The samples were filtered through 0.45-μm filters (Millipore, Billerica, MA, USA), followed by separation of tofacitinib on a reversed-phase column (AegisPak C_18_; 25 cm × 4.6 mm, 5 μm; Young Jin Biochrom, Seongnam, Korea). The mobile phase consisted of a 69.5:30.5 (v/v) mixture of 10 mM ammonium acetate buffer (pH 5.0) and acetonitrile, respectively, with a flow rate of 1.0 mL/min. Column effluent was monitored by a UV detector at 287 nm. The retention times of tofacitinib and the internal standard (hydrocortisone) were approximately 7.2 and 11.3 min, respectively. The lower limits of quantitation of tofacitinib in rat plasma, urine, and tissue homogenates were 0.01, 0.1, and 0.1 μg/mL, respectively, with intraday assay precision (coefficients of variation) in these samples being 3.69–5.88%, 4.21–6.18%, and 0.0205–8.74%, respectively. The interday assay precision was 5.06% for rat plasma and 5.46% for rat urine.

### 2.10. Pharmacokinetics Analysis

Pharmacokinetic parameters, including AUC, apparent volume of distribution at steady state (*V*_ss_), mean residence time (MRT), and time-averaged total body (CL), renal (CL_R_), and nonrenal (CL_NR_) clearances, were calculated by noncompartmental analysis (WinNonlin, Pharsight Corporation, Mountain View, CA, USA) using standard methods [15]. AUC values were calculated using the trapezoidal rule–extrapolation method [16]. The peak plasma concentration (*C*_max_) and time to reach *C*_max_ (*T*_max_) were obtained directly from the experimental data. The mean values of clearance [17], terminal half-life [18], and *V*_ss_ [19] were calculated using the harmonic mean method.

### 2.11. Statistical Analysis

All results are expressed as mean ± standard deviation (SD), except that *T*_max_ is expressed as median (range). Comparisons between two means were evaluated using Student’s *t*-tests and comparisons among three or more means by analysis of variance (ANOVA) with Tukey’s post-test. A *p* value <0.05 was considered statistically significant.

## 3. Results

### 3.1. Pharmacokinetics of Tofacitinib after Intravenous and Oral Administration to Rats

**Figure 2:**
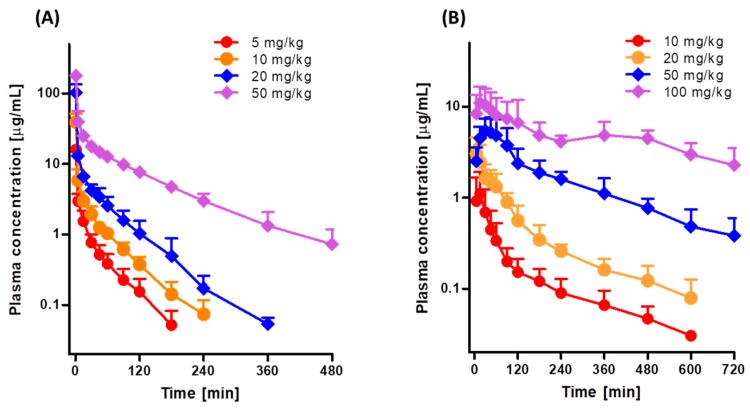
Mean arterial plasma concentration-time profiles of tofacitinib in Sprague-Dawley rats after (**A**) 1-min intravenous infusion of 5 (*n* = 9), 10 (*n* = 8), 20 (*n* = 7), and 50 (*n* = 7) mg/kg tofacitinib and (**B**) oral administration of 10 (*n* = 7), 20 (*n* = 8), 50 (*n* = 9), and 100 (*n* = 7) mg/kg tofacitinib. Bars represent standard deviations (SD).

Figure 2A shows the mean arterial plasma concentration-time profiles after intravenous infusion of 5, 10, 20, and 50 mg/kg tofacitinib over 1 min, and Table 1 shows the associated pharmacokinetic parameters. At all four doses, the mean arterial plasma concentrations of tofacitinib showed a polyexponential decrease. The dose-normalized AUCs following intravenous infusion of 5, 10, 20, and 50 mg/kg tofacitinib were 282, 342, 404, and 705 μg·min/mL, respectively (Table 1 and Supplementary Figure S1A). The dose-normalized AUC at 50 mg/kg was 2.50-, 2.06-, and 1.75-fold greater than the dose-normalized AUCs at 5, 10, and 20 mg/kg, respectively (*p* < 0.001 each). CL and CL_NR_ at 50 mg/kg were significantly slower than at 5, 10, and 20 mg/kg (*p* < 0.01 each). Thus, the terminal half-life and MRT were significantly longer following infusion of 50 mg/kg tofacitinib than the other doses (*p* < 0.001 each), whereas *V*_ss_ and the percentage of intravenous tofacitinib excreted unchanged in 24-h urine (*Ae*_0–24 h_) did not differ significantly among the four intravenous doses (Table 1). The CL_R_ was 69%, 71%, and 71% slower at 50 mg/kg than at 5, 10, and 20 mg/kg, respectively, because the *A_e_*_0–24 h_ was smaller and AUC was greater at 50 mg/kg than at the other doses. However, the percentages of tofacitinib recovered unchanged from the entire gastrointestinal tract (including its contents and feces) at 24 h (GI_24 h_) were negligible for all four intravenous doses (data not shown). Taken together, these findings indicate that the pharmacokinetic parameters of intravenous tofacitinib in rats were dependent on dose.

Figure 2B shows the mean arterial plasma concentration-time profiles following oral administration of 10, 20, 50, and 100 mg/kg tofacitinib, and Table 2 shows the associated pharmacokinetic parameters. Orally administered tofacitinib, at all four doses, was rapidly absorbed by the rat gastrointestinal tract, with tofacitinib detected in plasma within 5 min. After reaching *T*_max_, the plasma concentrations of tofacitinib showed a polyexponential decrease for all four doses. The dose-normalized AUCs following oral administration of 10, 20, 50, and 100 mg/kg tofacitinib were dose dependent, being 99.4, 135, 238, and 407 μg·min/mL, respectively (Table 2 and Supplementary Figure S1B). The dose-normalized AUC at 100 mg/kg was 4.09-, 3.01-, and 1.71-fold greater than those at 10, 20, and 50 mg/kg, respectively (*p* < 0.01 each). The CL_R_ was much slower at 100 mg/kg than at the other doses because AUC was significantly greater at 100 mg/kg. *T*_max_ was significantly longer at 100 mg/kg than at 10, 20 and 50 mg/kg (*p* < 0.05 each). The dose-normalized *C*_max_ (based on 10 mg/kg dose), GI_24 h_, and *Ae*_0–24 h_ did not differ significantly among the four oral doses studied (Table 2). Based on the AUC of 10 mg/kg intravenous tofacitinib, the *F* values for oral doses of 10, 20, 50, and 100 mg/kg were 29.1%, 39.3%, 69.7%, and 119%, respectively. These findings indicated that the pharmacokinetic parameters of orally administered tofacitinib in rats were dependent on dose.

### 3.2. Hepatic First-Pass Effect of Tofacitinib in Rats

**Figure 3:**
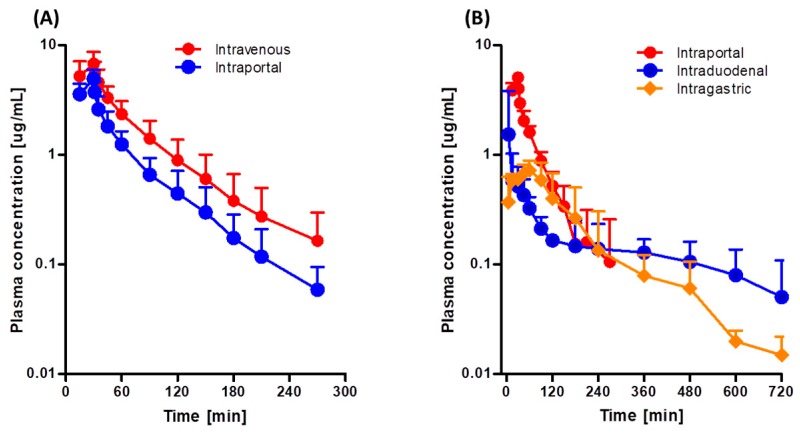
Mean arterial plasma concentration-time profiles of tofacitinib in Sprague-Dawley rats after (**A**) 30-min intravenous (*n* = 7) and intraportal (*n* = 7) infusions of 10 mg/kg tofacitinib and (**B**) 30-min intraportal (*n* = 6) infusion, and intraduodenal (*n* = 5) and intragastric (*n* = 5) instillations of 10 mg/kg tofacitinib. Bars represent standard deviations (SD).

Figure 3A shows the mean arterial plasma concentration-time profiles following intravenous and intraportal administration of 10 mg/kg tofacitinib, and Table 3 shows the associated pharmacokinetic parameters. The mean arterial plasma concentrations of tofacitinib administered intravenously and intraportally showed polyexponential reductions. The AUCs were 417 and 242 μg·min/mL, respectively, demonstrating considerable hepatic first-pass metabolism of tofacitinib after absorption into the portal vein, with 42.0% of the intravenous dose metabolized in the liver before entering the systemic circulation. As a result, the CL and CL_NR_ of tofacitinib were 67% and 60% faster, respectively, after intraportal administration. Furthermore, *V*_ss_ was 50% higher after intraportal than after intravenous administration of tofacitinib (*p* < 0.05).

### 3.3. Gastric and Intestinal First-Pass Effects of Tofacitinib in Rats

Figure 3B shows the mean arterial plasma concentration-time profiles following intragastric, intraduodenal, and intraportal administration of 10 mg/kg tofacitinib, and Table 4 shows the associated pharmacokinetic parameters. The AUCs of intragastrically and intraduodenally administered tofacitinib did not differ significantly (134 and 138 μg·min/mL), suggesting that the gastric first-pass effect of tofacitinib was negligible. In contrast, AUC was significantly lower after intraduodenal (138 μg·min/mL) than after intraportal (272 μg·min/mL) administration, indicating that the intestinal first-pass effect of tofacitinib was significant, with approximately 49.3% of the orally administered drug removed prior to entry into the portal vein.

### 3.4. Tissue Distribution of Tofacitinib in Rats

**Figure 4:**
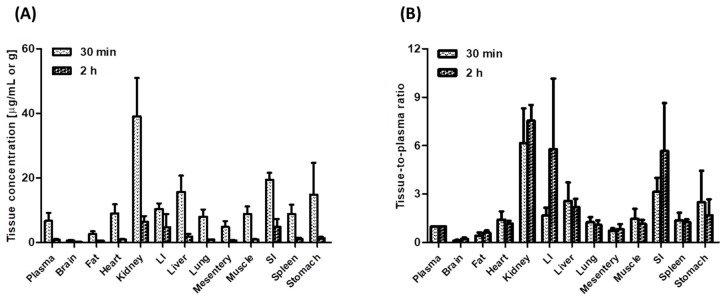
(**A**) Mean plasma and tissue/organ concentrations of tofacitinib and (**B**) tissue-to-plasma (T/P) ratios of tofacitinib 30 min (*n* = 4) and 2 h (*n* = 4) in Sprague-Dawley rats after 1-min intravenous infusion of 10 mg/kg tofacitinib. Data are expressed means ± standard deviations (SD). LI; large intestine, SI; small intestine.

Figure 4 shows the concentrations of tofacitinib in plasma (μg/mL) and in tissue samples (μg/g) and its tissue-to-plasma (T/P) ratios 30 min (distribution phase) and 2 h (elimination phase) after intravenous administration of 10 mg/kg tofacitinib. Tofacitinib was widely distributed in all rat tissues, with T/P ratios greater than 1.0 in every tissue except the brain, mesentery, and fat, both 30 min and 2 h after intravenous administration. At 30 min, tofacitinib was exclusively distributed in the kidneys, small intestine, and large intestine, with its concentrations remaining stable until 2 h after intravenous administration.

### 3.5. Biliary Excretion of Tofacitinib in Rats

Following a 1-min intravenous infusion of 10 mg/kg tofacitinib, less than 1% of the intravenous dose (0.703 ± 0.303%) was excreted in bile of each of the three rats studied, suggesting that biliary excretion of tofacitinib is a minor elimination pathway.

## 4. Discussion

The present study found that the dose-normalized AUC of tofacitinib was dependent on the administered dose. Plots of AUC versus dose for intravenous and oral tofacitinib yielded slopes of 2.74 and 4.65, respectively (Supplementary Figure S2). Several factors may account for the observed dose-dependent characteristics of tofacitinib. First, *V*_ss_ values did not differ significantly among the four intravenous doses, suggesting that the tofacitinib distribution process did not affect its dose-dependency. Thus, the contribution of *V*_ss_ to the dose-dependency of tofacitinib was negligible. Second, the contribution of CL_R_ to CL was not significant. *Ae*_0–24 h_ was less than 11.0% for all intravenous doses and less than 17.2% for all oral doses, with no significant differences among doses, suggesting that the contribution of renal excretion to the dose-dependent characteristics of tofacitinib is also low.

The renal extraction ratios (CL_R_/renal plasma flow rate) for urinary excretion of unchanged tofacitinib were estimated in rats based on its CL_R_, a reported renal blood flow rate of 36.8 mL/min/kg [20], and a hematocrit of approximately 45% [21]. The estimated renal extraction ratios following intravenous administration of 5, 10, 20, and 50 mg/kg tofacitinib were 13.3%, 13.8%, 13.8%, and 4.07%, respectively. These findings indicate that, in rats, tofacitinib has a low renal extraction ratio and that little tofacitinib is excreted via the kidneys. Therefore, most of the administered tofacitinib was eliminated via nonrenal pathways (CL_NR_).

The CL_NR_ of tofacitinib was affected by gastrointestinal (including biliary) excretion of unchanged drug and metabolic clearance. The contribution of gastrointestinal excretion to CL_NR_ was negligible, with no tofacitinib detected in the gastrointestinal tract 24 h after intravenous administration. The observed CL_NR_ of tofacitinib may therefore represent metabolic clearance of the drug, suggesting that changes in its CL_NR_ in rats may be due to changes in its metabolism. The increases in the dose-normalized AUC after intravenous and oral administration of tofacitinib may have been due to saturation of its metabolism, in agreement with the inverse relationship between slower CL_NR_ and higher intravenous dose. Oral tofacitinib also showed a dose-dependent AUC in humans, as evidenced by dose-dependent increases in dose-normalized AUCs [8,22].

Although the *F* values of tofacitinib differed in humans (74%) [7] and rats (29.1–33.8%), based on calculations using the same intravenous and oral doses, *F* values were not 100% in either species. Because it is difficult to measure first-pass metabolism in humans, first-pass metabolism in the liver and gastrointestinal tract was measured in rats. The *F* and GI_24 h_ of 10 mg/kg tofacitinib administered orally were 29.1% and 3.16%, respectively. The level of unchanged drug in the gastrointestinal tract (3.16%) may be due in part to the gastrointestinal (including biliary) excretion of absorbed drug. For comparison, the mean “true” fraction of unabsorbed dose (*F*_unabs_) following oral administration could be estimated using the equation [23];

GI_24 h, oral_ = *F*_unabs_ + (*F* × GI_24 h, intravenous_) (3)

where GI_24 h, oral_ and GI_24 h, intravenous_ are the percentages of oral and intravenous doses, respectively, remaining in the gastrointestinal tract after 24 h. Because GI_24 h, intravenous_ in the present study was negligible, *F*_unabs_ was almost equal to GI_24 h, oral_, indicating that gastrointestinal (including biliary) excretion of absorbed tofacitinib contributed little to the total drug recovered from the gastrointestinal tract after oral administration. Thus, approximately 96.8% of orally administered tofacitinib (10 mg/kg) was absorbed from the gastrointestinal tract in rats. Because only 3.16% of oral tofacitinib was not absorbed from the gastrointestinal tract at 24 h and the *F* value was 29.1%, approximately 67.7%·[100% − (3.16% + 29.1%)] of orally administered tofacitinib may have been eliminated by first-pass metabolism.

After intravenous administration of tofacitinib, the CL values of 14.5–36.3 mL/min/kg based on plasma data were considerably lower than the reported cardiac output of 296 mL/min/kg based on blood data [20] and a hematocrit of approximately 45% [21] in rats. These findings suggested that the first-pass effects of tofacitinib in the lungs and heart were negligible.

The AUCs were similar after intragastric and intraduodenal instillation of 10 mg/kg tofacitinib, suggesting that gastric first-pass effects on tofacitinib were negligible. However, the AUC after intraduodenal instillation of 10 mg/kg tofacitinib was 50.7% of that after intraportal administration, suggesting that approximately 49.3% of orally administered drug was not absorbed into the portal vein and that approximately 46.1%·[100% − (50.7% + 3.16%)] of orally administered tofacitinib was metabolized in the intestine before entering the portal vein. The AUC after intraportal administration of 10 mg/kg tofacitinib was 58.0% of that after intravenous administration, suggesting that the hepatic first-pass metabolism of tofacitinib after absorption into the portal vein was approximately 42.0%. Moreover, approximately 21.3% of the oral dose (42% of 50.7% of orally administered tofacitinib) was metabolized in the rat liver and 29.4% (50.7–21.3%) of the oral dose was absorbed into the systemic circulation. The latter percentage (29.4%) was close to the *F* value of 29.1%. Even though there is a species difference of bioavailability between human and rats, we could presume that 26%·(100–74%) of oral tofacitinib in humans was first-pass metabolized in the intestine and the liver with a similar ratio as rats. If a drug was not first-pass metabolized in the liver of the rat model, no hepatic first-pass metabolism was expected in humans. Considerable hepatic and intestinal first-pass metabolism has also been reported for other drugs, including ipriflavone [24], oltipraz [25], and sildenafil [26] in rats, and midazolam [27] in humans.

The AUCs were similar after intragastric and intraduodenal instillation of 10 mg/kg tofacitinib, suggesting that gastric first-pass effects on tofacitinib were negligible. However, the AUC after intraduodenal instillation of 10 mg/kg tofacitinib was 50.7% of that after intraportal administration, suggesting that approximately 49.3% of orally administered drug was not absorbed into the portal vein and that approximately 46.1%·[100% − (50.7% + 3.16%)] of orally administered tofacitinib was metabolized in the intestine before entering the portal vein. The AUC after intraportal administration of 10 mg/kg tofacitinib was 58.0% of that after intravenous administration, suggesting that the hepatic first-pass metabolism of tofacitinib after absorption into the portal vein was approximately 42.0%. Moreover, approximately 21.3% of the oral dose (42% of 50.7% of orally administered tofacitinib) was metabolized in the rat liver and 29.4% (50.7–21.3%) of the oral dose was absorbed into the systemic circulation. The latter percentage (29.4%) was close to the *F* value of 29.1%. Even though there is a species difference of bioavailability between human and rats, we could presume that 26%·(100–74%) of oral tofacitinib in humans was first-pass metabolized in the intestine and the liver with a similar ratio as rats. If a drug was not first-pass metabolized in the liver of the rat model, no hepatic first-pass metabolism was expected in humans. Considerable hepatic and intestinal first-pass metabolism has also been reported for other drugs, including ipriflavone [24], oltipraz [25], and sildenafil [26] in rats, and midazolam [27] in humans.

In humans, hepatic microsomal CYP3A4 and to a lesser extent CYP2C19 are involved in the metabolism of tofacitinib, oxidizing the pyrrolopyrimidine moiety and producing a carbonyl moiety, the major metabolite of tofacitinib [3]. CYP3A1(23)/2 and CYP2C11 are the main enzymes involved in drug metabolism in rats and are highly expressed in the rat liver and small intestine [28,29]. Human liver and intestinal CYP2C19 and rat CYP2C11 are highly homologous and human liver and gastrointestinal CYP3A4 and rat CYP3A1(23) share 73% homology [28,30]. We recently observed [31] that CYP3A1(23)/2 and CYP2C11 are the main CYPs responsible for the metabolism of tofacitinib in rats, as evidenced by a 46% greater AUC in rats pretreated with ketoconazole, an inhibitor of CYP3A1/2 [32], and a 39% greater AUC in rats pretreated with fluconazole, an inhibitor of CYP2C11 [33]. In contrast, the AUC of tofacitinib reduced by 56% in rats pretreated with dexamethasone, an inducer of CYP3A1/2 [34], and 26% in rats pretreated with rifampin, an inducer of CYP2C11 [35]. The AUCs of tofacitinib in rats pretreated with specific inhibitors or inducers of different CYP isoforms did not differ significantly [31]. Therefore, the dose dependent increases in AUCs of tofacitinib after intravenous and oral administration to rats suggested that hepatic first-pass metabolism of tofacitinib (42%) was saturated after intravenous administration, whereas its intestinal (46.1%) and/or hepatic (23.1%) first-pass metabolism was saturated after oral administration. This saturation may have been due to the saturable metabolism of tofacitinib by CYP3A1/2 and/or CYP2C11 in rat liver and intestine.

The distribution process of tofacitinib did not contribute to its dose-dependent profiles, as *V*_ss_ values did not differ significantly among the four intravenous doses. However, tofacitinib was widely distributed in rat tissues, especially in the small and large intestines, with the T/P ratios being higher for the intestines than for other tissues at both 30 min and 2 h. These findings suggest a mechanism for the effectiveness of tofacitinib in the treatment of ulcerative colitis, resulting in its approval in 2018 as the first oral drug for the treatment of chronic ulcerative colitis [6]. Tofacitinib is undergoing evaluation in clinical trials for the treatment of various diseases, including psoriasis [36,37], alopecia [38], atopic dermatitis [39], and ankylosing spondylitis [40].

Recently, several studies on tofacitinib pharmacokinetics were reported in patients with various diseases, including hepatic injury [41], renal failure [42], psoriasis [43], as well as inflammatory bowel disease [44], and most of them focused on the relationship between the drug concentration and the therapeutic efficacy. It was not well explained that the changes of plasma concentration according to diseases was related to the pharmacokinetic basis. In addition, pharmacokinetic drug interaction of tofacitinib is also expected since tofacitinib is mainly metabolized by CYP3A and is a substrate of P-glycoprotein [45]. Some pharmacokinetic drug interactions with tofacitinib were reported [46,47,48]. However, it is difficult to get the information from the clinical settings in order to evaluate the pharmacokinetic mechanism of the drug–disease or drug-drug interaction. Therefore, we need to further investigate the pharmacokinetic mechanism of the drug-disease or drug-drug interaction of tofacitinib in the rat model based on our pharmacokinetic characteristics of the drug in rats.

## 5. Conclusions

In conclusion, the low *F* of 10 mg/kg tofacitinib (29.1%) after oral administration to rats was mainly due to significant intestinal (46.1%) and hepatic (23.1%) first-pass metabolism. Our observation that the dose-normalized AUCs of tofacitinib in rats increased with increasing intravenous and oral doses, suggests that the hepatic and intestinal first-pass metabolism of tofacitinib was saturated by increasing its intravenous and oral doses.

## Figures and Tables

**Table 1:** Pharmacokinetic parameters of tofacitinib after 1-min intravenous infusion of the drug at various doses to male Sprague-Dawley rats. Data are expressed as mean ± standard deviation (SD).

Parameters.	5 mg/kg	10 mg/kg	20 mg/kg	50 mg/kg
	(*n* = 9)	(*n* = 8)	(*n* = 7)	(*n* = 7)
Body weight (g)	337 ± 39.2	347 ± 11.0	317 ± 15.9	356 ± 22.0
AUC (μg∙min/mL) ^a^	141 ± 23.0	342 ± 33.1	807 ± 146	3526 ± 572
Dose-normalized AUC (μg∙min/mL) ^a^	282 ± 46.0	342 ± 33.1	404 ± 73.0	705 ± 114
Terminal half-life (min) ^b^	38.2 ± 11.7	41.6 ± 7.86	48.1 ± 5.56	97.8 ± 19.2
MRT (min) ^b^	35.2 ± 11.4	41.1 ± 20.2	35.5 ± 7.50	105 ± 21.4
CL (mL/min/kg) ^c^	36.3 ± 6.02	29.5 ± 2.99	25.4 ± 4.26	14.5 ± 2.33
CL_R_ (mL/min/kg)	2.69 ± 1.70	2.80 ± 1.69	2.80 ± 1.17	0.824 ± 0.651
CL_NR_ (mL/min/kg) ^d^	32.9 ± 7.72	26.3 ± 2.16	22.6 ± 3.90	13.7 ± 2.12
*V*_ss_ (mL/kg)	1258 ± 460	1208 ± 579	900 ± 218	1489 ± 134
*Ae*_0–24 h_ (% of dose)	7.62 ± 5.08	9.40 ± 5.06	11.0 ± 3.72	5.55 ± 3.91

AUC values were normalized to tofacitinib dose of 10 mg/kg for statistical analysis. ^a^ 20 mg/kg was significantly different (*p* < 0.05) from 5 mg/kg. 50 mg/kg was significantly different (*p* < 0.001) from 5, 10 and 20 mg/kg. ^b^ 50 mg/kg was significantly different (*p* < 0.001) from 5, 10 and 20 mg/kg. ^c^ 5 mg/kg was significantly different from 10 (*p* < 0.05) and 20 (*p* < 0.001) mg/kg, respectively. 50 mg/kg was significantly different (*p* < 0.001) from 5, 10 and 20 mg/kg. ^d^ 5 mg/kg was significantly different from 10 (*p* < 0.05), 20 (*p* < 0.01) and 50 (*p* < 0.001) mg/kg, respectively. 50 mg/kg was significantly different from 10 (*p* < 0.001) and 20 (*p* < 0.01) mg/kg, respectively.

**Table 2:** Pharmacokinetic parameters of tofacitinib after oral administration of the drug at various doses to male Sprague-Dawley rats.

Parameters	10 mg/kg	20 mg/kg	50 mg/kg	100 mg/kg
	(*n* = 7)	(*n* = 8)	(*n* = 9)	(*n* = 7)
Body weight (g)	312 ± 19.8	308 ± 24.5	284 ± 22.3	286 ± 17.7
AUC (μg∙min/mL) ^a^	99.4 ± 35.5	269 ± 53.7	1192 ± 280	4073 ± 1787
Dose-normalized AUC (μg∙min/mL) ^a^	99.4 ± 35.5	135 ± 26.9	238 ± 56.0	407 ± 179
*C*_max_ (μg/mL)	1.13 ± 0.774	3.13 ± 1.27	5.62 ± 2.18	13.1 ± 5.48
*T*_max_ (min) ^b^	15.7 ± 7.32	15.7 ± 15.9	34.4 ± 26.7	170 ± 192
CL_R_ (mL/min/kg) ^c^	11.6 ± 2.87	6.97 ± 3.95	7.61 ± 2.82	3.37 ± 2.52
*Ae*_0–24 h_ (% of dose)	11.3 ± 4.60	10.0 ± 6.51	17.2 ± 3.98	13.0± 9.47
GI_24 h_ (% of dose)	3.16 ± 5.12	3.13 ± 3.27	2.77 ± 3.96	0.772± 0.905
*F* (%)	29.1	39.3	69.7	119

Data are expressed as mean ± standard deviation (SD). AUC and *C*_max_ values were normalized to tofacitinib dose of 10 mg/kg for statistical analysis. *F* was calculated by dose-normalized AUC (based on 10 mg/kg) after oral administration of tofacitinib divided by AUC after intravenous administration of the drug at dose of 10 mg/kg. ^a^ 10 mg/kg was significantly different (*p* < 0.05) from 50 mg/kg. 100 mg/kg was significantly different from 10 (*p* < 0.001), 20 (*p* < 0.001) and 50 (*p* < 0.01) mg/kg, respectively. ^b^ 100 mg/kg was significantly different (*p* < 0.05) from 10, 20 and 50 mg/kg. ^c^ 10 mg/kg was significantly different from 20 (*p* < 0.05) and 100 (*p* < 0.001) mg/kg, respectively.

**Table 3:** Pharmacokinetic parameters of tofacitinib after 30-min intravenous and intraportal infusion of the drug at dose of 10 mg/kg to male Sprague-Dawley rats.

Parameter	Intravenous	Intraportal
	(*n* = 7)	(*n* = 7)
Body weight (g)	292 ± 18.0	299 ± 28.6
AUC (μg∙min/mL)	417 ± 136	242 ± 66.0 ^**^
Terminal half-life (min)	46.2 ± 12.5	42.4 ± 10.4
MRT (min)	67.5 ± 26.8	59.6 ± 13.9
CL (mL/min/kg)	26.3 ± 8.85	43.8 ± 11.0 ^**^
CL_R_ (mL/min/kg)	2.34 ± 2.90	5.00 ± 4.69
CL_NR_ (mL/min/kg)	24.0 ± 6.46	38.5 ± 10.4 ^**^
*V*_ss_ (mL/kg)	1695 ± 737	2549 ± 720 ^*^
*Ae*_0–24 h_ (% of dose)	12.5 ± 5.20	10.7 ± 9.42

Data are expressed as mean ± standard deviation (SD). ^*^ *p* < 0.05; ^**^ *p* < 0.01.

**Table 4:** Pharmacokinetic parameters of tofacitinib after 30-min intraportal infusion, intraduodenal and intragastric instillation of the drug at dose of 10 mg/kg to male Sprague-Dawley rats.

Parameter	Intraportal	Intraduodenal	Intragastric
	(*n* = 6)	(*n* = 5)	(*n* = 5)
Body weight (g)	287 ± 10.6	282 ± 6.30	285 ± 4.97
AUC (μg∙min/mL) ^a^	272 ± 62.7	138 ± 33.2	134 ± 44.6
Terminal half-life (min)	44.8 ± 14.2		
*C*_max_ (μg/mL) ^b^	5.18 ± 0.586	1.55 ± 1.93	0.716 ± 0.255
*T*_max_ (min)	27.5 ± 6.12	38.8 ± 38.2	63.8 ± 18.9
MRT (min)	53.0 ± 22.3		
CL (mL/min/kg)	38.4 ± 8.47		
CL_R_ (mL/min/kg)	2.44 ± 1.23	5.72 ± 2.62	7.08 ± 2.91
CL_NR_ (mL/min/kg)	38.3 ± 6.16		
*V*_ss_ (mL/kg)	1900 ± 369		
*Ae*_0–24 h_ (% of dose)	5.82 ± 2.32	8.30 ± 5.10	8.90 ± 3.00

Data are expressed as mean ± standard deviation (SD). ^a^ Intraportal infusion was significantly different (*p* < 0.05) from intraduodenal and intragastric instillation. ^b^ Intraportal infusion was significantly different (*p* < 0.001) from intraduodenal and intragastric instillation.

## Supplementary Materials

The following are available online at https://www.mdpi.com/1999-4923/11/7/318/s1, **Figure S1:** Mean dose (mg/kg) versus dose-normalized AUC (μg·min/mL) of tofacitinib in Sprague-Dawley rats after (**A**) 1-min intravenous infusion of 5 (*n* = 9), 10 (*n* = 8), 20 (*n* = 7), and 50 (*n* = 7) mg/kg tofacitinib and (**B**) oral administration of 10 (*n* = 7), 20 (*n* = 8), 50 (*n* = 9), and 100 (*n* = 7) mg/kg tofacitinib. Bars represent standard deviations (SD). (**A**) 20 mg/kg was significantly different (*p* < 0.05) from5 mg/kg. 50 mg/kg was significantly different (*p* < 0.001) from 5, 10 and 20 mg/kg. (**B**) 10 mg/kg was significantly different (*p* < 0.05) from 50mg/kg. 100 mg/kg was significantly different from 10 (*p* < 0.001), 20 (*p* < 0.001) and 50 (*p* < 0.01) mg/kg, respectively, **Figure S2:** Plots of dose versus AUC of tofacitinib in Sprague-Dawley rats after (**A**) 1-min intravenous infusion of 5, 10, 20, and 50 mg/kg tofacitinib and (**B**) oral administration of 10, 20, 50, and 100 mg/kg tofacitinib. Dose and AUC ratios were calculated based on 5 and 10 mg/kg dose and respective AUC for intravenous and oral administration, respectively.
