# Supplementary material for: Dose-Dependent Pharmacokinetics of Tofacitinib in Rats: Influence of Hepatic and Intestinal First-Pass Metabolism
Source: Pharmaceutics. 2019 Jul 5;11(7):318. doi: 10.3390/pharmaceutics11070318 (PMC6681021; doi:10.3390/pharmaceutics11070318)
Supplement: Supplementary file 1 [file pharmaceutics-11-00318-s001.pdf]

pharmaceutics

MDPI

Supplementary Materials: Dose-Dependent Pharmacokinetics of Tofacitinib in Rats: Influence of Hepatic and Intestinal First-Pass Metabolism

Ji Sang Lee and So Hee Kim *

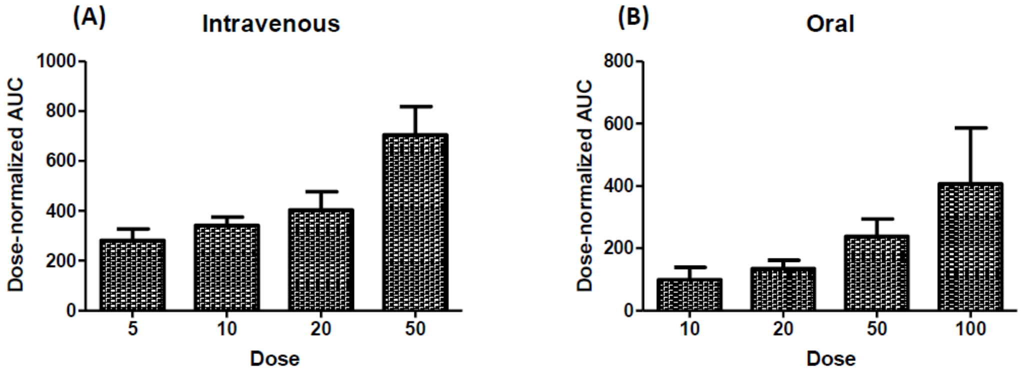

Figure S1. Mean dose (mg/kg) versus dose-normalized AUC (μg·min/mL) of tofacitinib in Sprague-Dawley rats after (**A**) 1-min intravenous infusion of 5 (n = 9), 10 (n = 8), 20 (n = 7), and 50 (n = 7) mg/kg tofacitinib and (**B**) oral administration of 10 (n = 7), 20 (n = 8), 50 (n = 9), and 100 (n = 7) mg/kg tofacitinib. Bars represent standard deviations (SD). (**A**) 20 mg/kg was significantly different ($p < 0.05$) from5 mg/kg. 50 mg/kg was significantly different ($p < 0.001$) from 5, 10 and 20 mg/kg. (**B**) 10 mg/kg was significantly different ($p < 0.05$) from 50mg/kg. 100 mg/kg was significantly different from 10 ($p < 0.001$), 20 ($p < 0.001$) and 50 ($p < 0.01$) mg/kg, respectively.

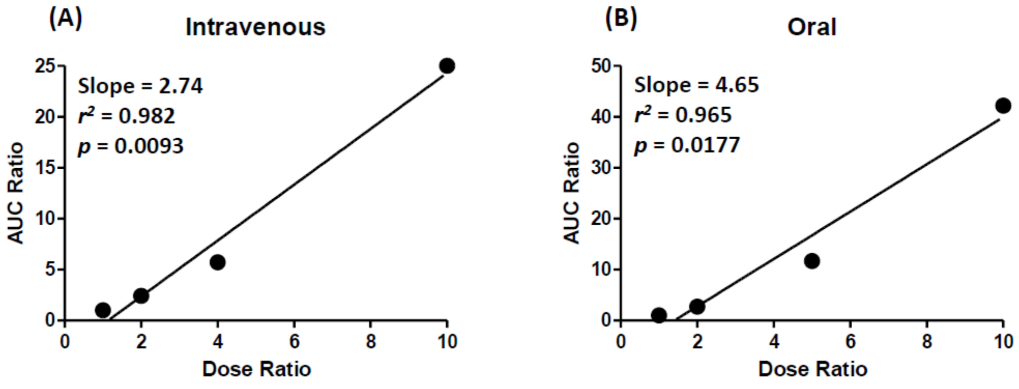

Figure S2. Plots of dose versus AUC of tofacitinib in Sprague-Dawley rats after (**A**) 1-min intravenous infusion of 5, 10, 20, and 50 mg/kg tofacitinib and (**B**) oral administration of 10, 20, 50, and 100 mg/kg tofacitinib. Dose and AUC ratios were calculated based on 5 and 10 mg/kg dose and respective AUC for intravenous and oral administration, respectively.
